# Risk factors for pancreatitis following endoscopic retrograde cholangiopancreatography

**DOI:** 10.1002/bjs5.50162

**Published:** 2019-04-02

**Authors:** E. Syrén, S. Eriksson, L. Enochsson, A. Eklund, G. Sandblom

**Affiliations:** ^1^ Department of Surgical Sciences Uppsala University Uppsala Sweden; ^2^ Department of Surgery Uppsala University Hospital Uppsala Sweden; ^3^ Department of Surgery, Centre for Clinical Research Västmanland Regional Hospital Västerås Sweden; ^4^ Department of Surgical and Perioperative Sciences, Sunderby Research Unit Umeå University Umeå Sweden; ^5^ Department of Clinical Science and Education Södersjukhuset Karolinska Institutet Stockholm Sweden; ^6^ Department of Surgery Södersjukhuset Stockholm Sweden

## Abstract

The aim of this study was to assess whether sex, age, ASA grade, previous history of acute pancreatitis, diabetes, hyperlipidaemia, hypercalcaemia, kidney disease and liver cirrhosis influence the risk for developing post‐endoscopic retrograde cholangiopancreatography (ERCP) pancreatitis (PEP). A total of 15 800 ERCP procedures retrieved from the Swedish National Quality Register for Gallstone Surgery and ERCP (GallRiks) for 2006–2014 were identified and cross‐checked with the National Patient Register. Women, patients aged less than 65 years, patients with hyperlipidaemia and those with a previous history of acute pancreatitis had a significantly increased risk of PEP, whereas patients with diabetes had a significantly decreased risk.

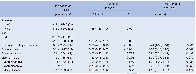

Risk of pancreatitis following ERCP

## Abstract (sv)

**Background:**

The risk of post‐endoscopic retrograde cholangiopancreatography (ERCP) pancreatitis (PEP) could be related to technical or patient‐related factors. The aim of this study was to assess whether clinical variables and co‐morbidities influence the risk of developing PEP.

**Methods:**

Data were retrieved from the Swedish GallRiks registry, including all ERCP procedures performed in 2006–2014 for common bile duct stones. A total of 15 800 procedures were identified and cross‐checked. Univariable and multivariable logistic regression analyses were conducted with the endpoint of PEP using the following co‐variables: age, sex, ASA grade, previous history of acute pancreatitis, diabetes, hyperlipidaemia, hypercalcaemia, kidney disease and liver cirrhosis.

**Results:**

Women (odds ratio (OR) 1·33, 95 per cent c.i. 1·14 to 1·55), patients aged less than 65 years (OR 1·68, 1·45 to 1·94), patients with hyperlipidaemia (OR 1·32, 1·02 to 1·70) and those with a previous history of acute pancreatitis (OR 5·44, 4·68 to 6·31) had a significantly increased risk of PEP. In a subgroup analysis of patients with a previous history of acute pancreatitis, the mean time from previous pancreatitis to ERCP was 4423 days in patients who developed PEP *versus* 6990 days in patients who did not (*P* = 0·037). However, when the previous episode of pancreatitis had occurred more than 30 days before ERCP, this association was no longer significant (*P* = 0·858). Patients with diabetes had a decreased risk of PEP (OR 0·64, 0·48 to 0·85).

**Conclusion:**

Age, sex, hyperlipidaemia and previous history of recent acute pancreatitis increase the risk of PEP. The reduced risk of PEP in patients with diabetes should be explored in future studies.

## Introduction

One of the most feared complications described after endoscopic retrograde cholangiopancreatography (ERCP) is post‐ERCP pancreatitis (PEP), which occurs with an incidence of 3·5–5 per cent^1^, ^2^. PEP is defined^3^ as ‘clinical pancreatitis with amylase at least three times the upper limit of normal at more than 24 h after the procedure requiring hospital admission or prolongation of planned admission’, whereas its severity has been based mainly on the length of hospital stay.

The risk of developing PEP can be assessed in relation to several variables, including technical factors (manipulation and injection of contrast into the pancreatic duct, cannulation attempts lasting more than 5 min, and biliary balloon sphincter dilatation) and patient‐related factors such as female sex, younger age, sphincter of Oddi dysfunction^2^, ^3^, ^4^, ^5^ and a previous history of PEP or pancreatitis^6^. The most common causes of acute pancreatitis are biliary stone and alcohol abuse. However, other conditions, including long‐term haemodialysis or peritoneal dialysis, are associated with an increased risk^7^, ^8^, and co‐morbidities such as peptic ulcer, hepatic disease and diabetes are frequently described^9^.

In particular, patients with type 2 diabetes have a 1·91‐fold increased risk of developing biliary disease and a 2·83‐fold increased risk of pancreatitis^10^. An increased risk of pancreatitis has also been shown to be associated with younger age and the presence of hypertriglyceridaemia^11^, and a reduced risk associated with the use of insulin and long‐term use of metformin in diabetic patients^12^. Finally, patients with more advanced cirrhosis (Child–Pugh grade B and C) have a higher incidence of ERCP complications than those with Child–Pugh grade A^13^, and an increased risk of postprocedure bleeding, although not of PEP^14^.

The aim of the present study was to investigate the risk of PEP in patients with diabetes, liver cirrhosis, hyperlipidaemia, hypercalcaemia and kidney disease.

## Methods

Data in the GallRiks registry (the Swedish National Quality Register for Gallstone Surgery and ERCP) were retrieved and reviewed. GallRiks was started in 2005 and includes approximately 90 per cent of cholecystectomies and ERCPs performed in Sweden. GallRiks is regularly externally validated, and the validation process and its national coverage results are published each year^15^, ^16^, ^17^. Records include patient‐ and procedure‐related data as well as intraoperative and postoperative complications up to 30 days after ERCP.

For the present study, all ERCP procedures registered in GallRiks between 2006 and 2014 for bile duct stones were included. ERCPs conducted for other indications, repeated ERCP (in the same patient) and ERCPs with missing follow‐up data were excluded.

PEP was defined as typical abdominal pain, a serum amylase level more than three times the upper limit of normal more than 24 h after ERCP, and the need for hospitalization^3^.

Data on chronic disease (diabetes, liver cirrhosis, hyperlipidaemia, hypercalcaemia and kidney disease) and previous episodes of acute pancreatitis were obtained by cross‐checking GallRiks data with that in the National Patient Register using ICD codes (*Table* 1).

**Table 1:** ICD codes for the different conditions

	ICD9	ICD10
Acute pancreatitis		K85
Diabetes (all)	250	E10
	E11
		E12
Diabetes type 1		E10
Liver cirrhosis	456C	I85
	571	K70.3
		K71.7
		K74
Hyperlipidaemia		E78
Hypercalcaemia		E83.5
Kidney disease	402A	I12.0
	402B	I13.1
	403B	N03.2–N03.7
	403X	N05.2–N05.7
	582	N19
	583A–583H	N25.0
	585	Z49.0–Z49.2
	586	Z94.0
	588A	Z99.2
	V42A	
	V45B	
	V56	

The Regional Ethics Review Board in Stockholm approved the study (reference number 2015/339‐31/1).

### Statistical analysis

Univariable and multivariable logistic regression analyses with the endpoint of PEP were performed. In the multivariable analyses, adjustment was made for sex and age (at least 65 years *versus* less than 65 years). Adjustments in the multivariable analysis were made based on assumptions of cause–effect relationships.

A subgroup analysis was conducted in patients with a previous history of pancreatitis. The mean(s.d.) time between the previous episode of pancreatitis and ERCP was determined and compared in patients who developed PEP following ERCP and those who did not have this complication, using Student's *t* test. Statistical analysis was performed with SPSS® version 25 (IBM, Armonk, New York, USA).

## Results

**Figure 1:**
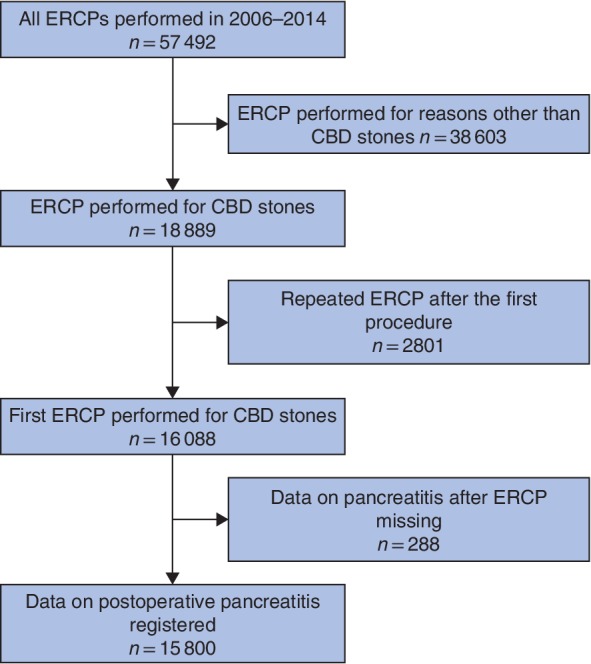
Flow diagram for the study. ERCP, endoscopic retrograde cholangiopancreatography; CBD, common bile duct

Some 15 800 of 57 492 ERCP procedures carried out between 2006 and 2014 that met the study design criteria were analysed (*Fig*. 1). Patient characteristics and risk factors for PEP are shown in *Table* 2.

**Table 2:** Baseline characteristics of patients with pancreatitis after endoscopic retrograde cholangiopancreatography registered in the Swedish Nationwide Data Register GallRiks, 2006–2014

	No. of patients (*n* = 15 800)
Age (years)*	64·6(19·1)
Sex	
M	6140 (38·9)
F	9660 (61·1)
ASA fitness grade	
I	5208 (33·0)
II	7484 (47·4)
III	2944 (18·6)
IV	163 (1·0)
V	1 (0·0)
History of acute pancreatitis	2567 (16·2)
Diabetes	1947 (12·3)
Hyperlipidaemia	1394 (8·8)
Hypercalcaemia	58 (0·4)
Kidney disease	579 (3·7)
Liver cirrhosis	185 (1·2)

Values in parentheses are percentages unless indicated otherwise; *values are mean(s.d.).

*Table* 3 shows the results of univariable and multivariable analyses with the endpoint of PEP. Univariable analysis found a significantly greater risk of PEP in women (odds ratio (OR) 1·33, 95 per cent c.i. 1·14 to 1·55), patients aged less than 65 years (OR 1·68, 1·45 to 1·94) and those with a previous history of acute pancreatitis (OR 5·26, 4·53 to 6·10). Patients with diabetes had a lower risk of PEP (OR 0·55, 0·42 to 0·72). In multivariable analysis, after adjustment for age and sex, a previous history of acute pancreatitis (OR 5·44, 4·68 to 6·31) and hyperlipidaemia (OR 1·32, 1·02 to 1·70) were found to increase the risk of PEP, whereas diabetes decreased the risk (OR 0·64, 0·48 to 0·85).

**Table 3:** Univariable and multivariable logistic analysis of risk factors for pancreatitis after endoscopic retrograde cholangiopancreatography

		Univariable analysis		Multivariable analysis‡	
	Incidence of post‐ERCP pancreatitis*	Odds ratio†	*P*	Odds ratio†	*P*
Age (years)					
≥ 65	349 of 9140 (3·8)				
< 65	416 of 6660 (6·2)	1·68 (1·45, 1·94)	< 0·001		
Sex					
M	250 of 6140 (4·1)				
F	515 of 9660 (5·3)	1·33 (1·14, 1·55)	< 0·001		
History of acute pancreatitis	363 of 2567 (14·1)	5·26 (4·53, 6·10)	< 0·001	5·44 (4·68, 6·31)	< 0·001
Diabetes (all)	56 of 1947 (2·9)	0·55 (0·42, 0·72)	< 0·001	0·64 (0·48, 0·85)	0·002
Diabetes type 1	21 of 564 (3·7)	0·72 (0·47, 1·13)	0·724	0·84 (0·54, 1·31)	0·437
Liver cirrhosis	12 of 185 (6·5)	1·37 (0·76, 2·47)	0·296	1·39 (0·77, 2·51)	0·277
Hyperlipidaemia	72 of 1394 (5·2)	1·08 (0·84, 1·38)	0·556	1·32 (1·02, 1·70)	0·036
Hypercalcaemia	2 of 58 (3·4)	0·70 (0·17, 2·88)	0·622	0·76 (0·18, 3·11)	0·756
Kidney disease	27 of 579 (4·7)	0·96 (0·65, 1·42)	0·838	1·16 (0·78, 1·72)	0·474

Values in parentheses are *percentages and †95 per cent confidence intervals. ‡Adjustments were made for sex and age (at least 65 years *versus* less than 65 years). ERCP, endoscopic retrograde cholangiopancreatography.

In a subgroup analysis of 2567 patients with a previous history of acute pancreatitis, the mean(s.d.) time from the previous episode of pancreatitis to ERCP was 4423(5262) days in patients who developed PEP *versus* 6990(5071) days in those who did not develop PEP (*P* = 0·037). However, when the previous episode of pancreatitis had occurred more than 30 days before ERCP, this association was no longer significant. In that group, the mean time from pancreatitis to ERCP was 7772(4747) days in patients who did not develop PEP and 7727(4781) days in those who did (*P* = 0·858).

## Discussion

This national register‐based analysis found that women, patients aged less than 65 years and those with a previous history of acute pancreatitis had a significantly greater risk of PEP, as documented previously by other authors^2^, ^3^, ^4^, ^5^, ^6^. However, as it is difficult to distinguish a new episode of acute pancreatitis from an exacerbation of an ongoing process, patients with pancreatitis immediately before ERCP were excluded, indicating that an episode of pancreatitis occurring more than 30 days before elective ERCP had no association with the development of PEP.

In accordance with previous studies^11^, ^18^ investigating hypertriglyceridaemia, hyperlipidaemia was also found to increase the risk of PEP. However, other associated co‐morbidities such as obesity were not investigated in the present study as data on BMI were not available in the registry. Similarly, other possible conditions influencing the risk of PEP, such as alcohol abuse and medications, are not registered consistently in GallRiks.

Although the literature^7^, ^8^, ^19^ documents contrasting results with respect to hypercalcaemia/kidney disease and risk of PEP, it should be noted that only 58 patients in the present cohort had hypercalcaemia and 579 had kidney disease, with no data on the degree of renal failure; thus it would be difficult to draw any firm conclusion regarding the association between hypercalcaemia/kidney disease and PEP.

Similar to previous findings^13^, ^14^, liver cirrhosis was not found to be a risk factor for PEP.

In contrast to previous studies^10^, ^20^, in which diabetes was shown to be associated with acute pancreatitis, a decreased risk of PEP was found in diabetic patients. This was confirmed in the multivariable analysis, after adjustment for age and sex. It has been shown previously^12^ that the risk of acute pancreatitis is dependent on the type of diabetes medication received by patients. Although the cohort of diabetic patients consisted of patients on different kinds of diabetic treatment, the registry lacked information on disease severity and treatment; thus these associations were not investigated and need to be validated in future studies.

## Disclosure

The authors declare no conflict of interest.

## References

[1] GallRiks . [Annual Report 2016]; 2017 http://www.ucr.uu.se/gallriks/fou/arsrapporter [accessed 31 May 2018].

[2] Dumonceau JM , Andriulli A , Elmunzer BJ , Mariani A , Meister T , Deviere J *et al*; European Society of Gastrointestinal Endoscopy . Prophylaxis of post‐ERCP pancreatitis: European Society of Gastrointestinal Endoscopy (ESGE) guideline – updated June 2014. *Endoscopy* 2014; 46: 799–815. 10.1055/s-0034-1377875 25148137

[3] Cotton PB , Lehman G , Vennes J , Geenen JE , Russell RC , Meyers WC *et al* Endoscopic sphincterotomy complications and their management: an attempt at consensus. Gastrointest Endosc 1991; 37: 383–393. 2070995 10.1016/s0016-5107(91)70740-2

[4] Freeman ML . Post‐ERCP pancreatitis: patient and technique‐related risk factors. JOP 2002; 3: 169–176. 12432183

[5] Pezzilli R , Romboli E , Campana D , Corinaldesi R. Mechanisms involved in the onset of post‐ERCP pancreatitis. JOP 2002; 3: 162–168. 12432182

[6] Chen JJ , Wang XM , Liu XQ , Li W , Dong M , Suo ZW *et al* Risk factors for post‐ERCP pancreatitis: a systematic review of clinical trials with a large sample size in the past 10 years. Eur J Med Res 2014; 19: 26. 24886445 10.1186/2047-783X-19-26 PMC4035895

[7] Hou SW , Lee YK , Hsu CY , Lee CC , Su YC . Increased risk of acute pancreatitis in patients with chronic hemodialysis: a 4‐year follow‐up study. PLoS One 2013; 8: e71801. 23977145 10.1371/journal.pone.0071801 PMC3748083

[8] Chen HJ , Wang JJ , Tsay WI , Her SH , Lin CH , Chien CC . Epidemiology and outcome of acute pancreatitis in end‐stage renal disease dialysis patients: a 10‐year national cohort study. Nephrol Dial Transplant 2017; 32: 1731–1736. 28088773 10.1093/ndt/gfw400

[9] Shen HN , Lu CL , Li CY . Epidemiology of first‐attack acute pancreatitis in Taiwan from 2000 through 2009: a nationwide population‐based study. Pancreas 2012; 41: 696–702. 22699142 10.1097/MPA.0b013e31823db941

[10] Noel RA , Braun DK , Patterson RE , Bloomgren GL . Increased risk of acute pancreatitis and biliary disease observed in patients with type 2 diabetes: a retrospective cohort study. Diabetes Care 2009; 32: 834–838. 19208917 10.2337/dc08-1755 PMC2671118

[11] Albai O , Roman D , Frandes M . Hypertriglyceridemia, an important and independent risk factor for acute pancreatitis in patients with type 2 diabetes mellitus. Ther Clin Risk Manag 2017; 13: 515–522. 28450786 10.2147/TCRM.S134560 PMC5399973

[12] Gonzalez‐Perez A , Schlienger RG , Rodríguez LA . Acute pancreatitis in association with type 2 diabetes and antidiabetic drugs: a population‐based cohort study. Diabetes Care 2010; 33: 2580–2585. 20833867 10.2337/dc10-0842 PMC2992194

[13] Adler DG , Haseeb A , Francis G , Kistler CA , Kaplan J , Ghumman SS *et al* Efficacy and safety of therapeutic ERCP in patients with cirrhosis: a large multicenter study. Gastrointest Endosc 2016; 83: 353–359. 26297868 10.1016/j.gie.2015.08.022

[14] Navaneethan U , Njei B , Zhu X , Kommaraju K , Parsi MA , Varadarajulu S . Safety of ERCP in patients with liver cirrhosis: a national database study. Endosc Int Open 2017; 5: E303–E314. 28393104 10.1055/s-0043-102492 PMC5383432

[15] GallRiks . *Homepage* www.ucr.uu.se/gallriks/ [accessed 18 March 2019].

[16] Rystedt J , Montgomery A , Persson G. Completeness and correctness of cholecystectomy data in a national register – GallRiks. Scand J Surg 2014; 103: 237–244. 24737852 10.1177/1457496914523412

[17] Enochsson L , Thulin A , Osterberg J , Sandblom G , Persson G . The Swedish Registry of Gallstone Surgery and Endoscopic Retrograde Cholangiopancreatography (GallRiks): a nationwide registry for quality assurance of gallstone surgery. JAMA Surg 2013; 148: 471–478. 23325144 10.1001/jamasurg.2013.1221

[18] Fortson MR , Freedman SN , Webster PD 3rd . Clinical assessment of hyperlipidemic pancreatitis. Am J Gastroenterol 1995; 90: 2134–2139. 8540502

[19] Bai HX , Giefer M , Patel M , Orabi AI , Husain SZ . The association of primary hyperparathyroidism with pancreatitis. J Clin Gastroenterol 2012; 46: 656–661. 22874807 10.1097/MCG.0b013e31825c446c PMC4428665

[20] Shen HN , Lu CL , Li CY . Effect of diabetes on severity and hospital mortality in patients with acute pancreatitis: a national population‐based study. Diabetes Care 2012; 35: 1061–1066. 22446175 10.2337/dc11-1925 PMC3329843

